# Benchmarking pH-field coupled microkinetic modeling against oxygen reduction in large-scale Fe–azaphthalocyanine catalysts†

**DOI:** 10.1039/d4sc00473f

**Published:** 2024-03-15

**Authors:** Di Zhang, Yutaro Hirai, Koki Nakamura, Koju Ito, Yasutaka Matsuo, Kosuke Ishibashi, Yusuke Hashimoto, Hiroshi Yabu, Hao Li

**Affiliations:** a Advanced Institute for Materials Research (WPI-AIMR), Tohoku University Sendai 980-0811 Japan hiroshi.yabu.d5@tohoku.ac.jp li.hao.b8@tohoku.ac.jp; b AZUL Energy, Inc. 1-9-1, Ichibancho, Aoba-Ku Sendai 980-0811 Japan; c Research Institute for Electronic Science (RIES), Hokkaido University N21W10 Sapporo 001-0021 Japan; d Tohoku Forum for Creativity, Tohoku University Sendai 980-8577 Japan

## Abstract

Molecular metal–nitrogen–carbon (M–N–C) catalysts with well-defined structures and metal-coordination environments exhibit distinct structural properties and excellent electrocatalytic performance, notably in the oxygen reduction reaction (ORR) for fuel cells. Metal-doped azaphthalocyanine (AzPc) catalysts, a variant of molecular M–N–Cs, can be structured with unique long stretching functional groups, which make them have a geometry far from a two-dimensional geometry when loaded onto a carbon substrate, similar to a “dancer” on a stage, and this significantly affects their ORR efficiency at different pH levels. However, linking structural properties to performance is challenging, requiring comprehensive microkinetic modeling, substantial computational resources, and a combination of theoretical and experimental validation. Herein, we conducted pH-dependent microkinetic modeling based upon *ab initio* calculations and electric field-pH coupled simulations to analyze the pH-dependent ORR performance of carbon-supported Fe–AzPcs with varying surrounding functional groups. In particular, this study incorporates large molecular structures with complex long-chain “dancing patterns”, each featuring >650 atoms, to analyze their performance in the ORR. Comparison with experimental ORR data shows that pH-field coupled microkinetic modeling closely matches the observed ORR efficiency at various pH levels in Fe–AzPc catalysts. Our results also indicate that assessing charge transfer at the Fe-site, where the Fe atom typically loses around 1.3 electrons, could be a practical approach for screening appropriate surrounding functional groups for the ORR. This study provides a direct benchmarking analysis for the microkinetic model to identify effective M–N–C catalysts for the ORR under various pH conditions.

## Abstract 2

Molecular Fe azaphthalocyanine catalysts with diverse long-chain “dancing patterns” demonstrate variable oxygen reduction reaction performance for fuel cells, aligning well with our pH-field coupled microkinetic model.

## Graphical abstract

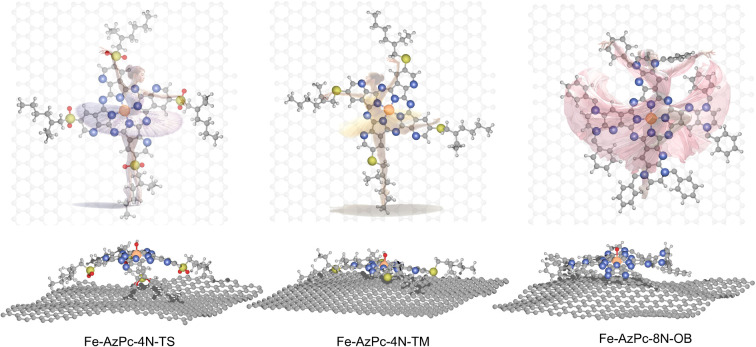

## Introduction

The electrocatalytic oxygen reduction reaction (ORR) is a pivotal process occurring at the cathode of hydrogen fuel cells and is essential for the efficient conversion of green hydrogen into electricity.^1,2^ This process, however, predominantly relies on expensive platinum-based materials for catalysis. Additionally, the substantial overpotential associated with these materials also poses significant challenges. Despite considerable efforts to explore a range of materials for the ORR, many catalysts continue to face the challenges of high costs, suboptimal performance, and short lifetime.^3,4^ Developing non-precious metal ORR catalysts and highly precise structure–performance catalytic models remains an urgent priority.

As effective alternatives to precious metal-based catalysts, emerging Pt-free metal–nitrogen–carbon (M–N–C) catalysts, *e.g*., Fe-based M–N–C catalysts, are a class of promising catalysts for the ORR, with clear advantages such as low cost, high performance, and tunable selectivity.^5–7^ Conventionally, many M–N–C catalysts were experimentally prepared by pyrolysis methods at high temperatures, *e.g*., 700–1300 K,^8^ which would result in a variety of coordination environments of active metal centers and the uncontrollable formation of defects.^9,10^ This has, inevitably, led to a big challenge in understanding the structure–performance relationships of M–N–Cs for the ORR. In recent years, there have been many debates regarding the active sites and the active coordination environments of the metals anchored at the N–C substrate.^10–13^ Though theoretical calculations (primarily based on density functional theory, DFT) can fill in some knowledge gaps using reaction-free energy calculations and microkinetic modeling,^14,15^ there are too many possible active sites/environments that need to be analyzed. Meanwhile, due to the lack of a “standard answer” from the structure–performance relationships resulting from pyrolysis methods in synthesis, it is difficult to perform a precise benchmarking analysis between theory and experiments to evaluate the accuracy of theoretical calculations and modeling.

In contrast, molecular M–N–C catalysts, achieved by doping metal elements onto structurally well-defined organic molecules, offer well-defined metal-coordination environments. This unique feature provides an ideal platform for directly comparing theoretical computations with experimental outcomes, thereby facilitating the precise determination of the accuracy of microkinetic modeling in catalysis.^10,16^ Besides, compared to conventional M–N–C catalysts, molecular M–N–C catalysts sometimes perform very differently in the ORR in terms of activity and reaction selectivity,^17^ and meanwhile, their performance can be tuned by adjusting the surrounding functional groups of the molecule species.^16^ Therefore, understanding the intricate structure–performance relationships inherent in molecular M–N–C catalysts is crucial for the tailored design of these catalysts for specific functions. Driven by these insights, conducting a benchmarking analysis that bridges theory and experiments with molecular M–N–C catalysts emerges as a task of profound scientific importance.

**Fig. 1:**
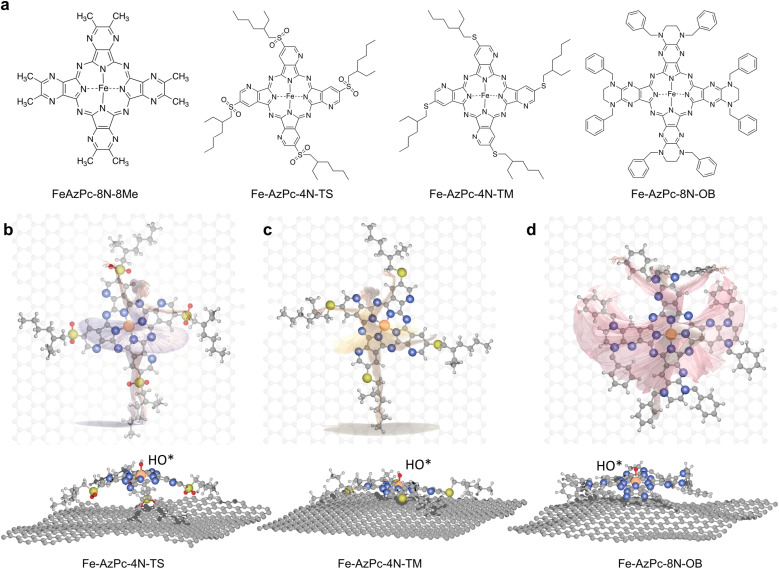
Structures of long-chain Fe–azaphthalocyanine (AzPc) molecular catalysts. (a) Chemical structures of Fe–AzPc–8N–8Me (Me = methyl), Fe–AzPc–4N–TS (TS = tetrakis sulfonyl), Fe–AzPc–4N–TM (TM = tetrakis 2-ethylhexyl thio), and Fe–AzPc–8N–OB (OB = octakis phenylmethyl). After DFT geometric relaxations, different “dancing patterns” emerged due to the varying interactions between the molecular side chains and the graphene substrate. These include (b) Fe–AzPc–4N–TS, (c) Fe–AzPc–4N–TM, and (d) Fe–AzPc–4N–OB, resembling molecules dancing on the ‘stage’ of graphene. Orange, gray, blue, red, yellow, and white spheres represent Fe, C, N, O, S, and H, respectively.

As a type of molecular M–N–C catalyst, metal azaphthalocyanine derivative (M–AzPc) electrocatalysts can be synthesized from economical pigments and Ketjen black without the need for conventional high-temperature pyrolysis.^18–20^ Moreover, by avoiding high-temperature pyrolysis, the coordination environments around the metal atoms within the AzPc derivatives can be more precisely controlled. A typical AzPc structure contains four pyrrole-like N that can coordinate with the metal atom, with the surrounding functional groups that can be well constructed by specific synthetic treatments. Fig. 1a shows long-chain Fe–AzPc structures which will be analyzed in this paper, namely, Fe–AzPc–8N–8Me (Me = methyl), Fe–AzPc–4N–TS (TS = tetrakis sulfonyl), Fe–AzPc–4N–TM (TM = tetrakis 2-ethylhexyl thio), and Fe–AzPc–8N–OB (OB = octakis phenylmethyl). These structures have more than 650 atoms and exhibit unique long-stretching functional groups surrounding the central area of a molecule. After DFT structural relaxation, these structures are stereoscopic and far from a two-dimensional structure when loaded onto a carbon substrate for electrocatalysis, with the geometries like a “dancer” (Fig. 1b and c). These “dancing patterns” can hardly be seen on the molecular M–N–C catalysts without any long stretching functional groups (see Fig. S2† for more details) and may lead to different electronics of the ORR active center (*e.g*., the Fe-site), which in turn leads to different ORR performances.

Motivated by the above, herein, we conducted pH-dependent microkinetic modeling analysis based upon spin-polarized DFT with van der Waals interactions (DFT-D3) and electric field-pH coupled simulations to analyze the pH-dependent ORR performance of typical carbon-supported Fe–AzPcs with varying surrounding functional groups (Fig. 1), and compared these results with subsequent ORR experiments using these Fe–AzPc catalysts. We found that although these Fe–AzPcs have the same active center, different “dancing patterns” lead to very different ORR performances due to the variety in the surrounding functional groups, which is attributed to the change of the electron number of the Fe-site induced by different degrees of the structural relaxation of a molecule. Our results show that the pH-field coupled microkinetic modeling method for the ORR can lead to good agreement with experimental observation at different pH, which can provide precise predictive insights into the ORR activities of M–N–C catalysts.

## Methods

### Binding free energy calculations

In this study, the Vienna *ab initio* simulation package (VASP) was employed for conducting density functional theory (DFT) calculations. These calculations were pivotal in determining the binding energies of ORR adsorbates, crucial for establishing scaling relations. The electronic exchange and correlations were meticulously described using the revised Perdew–Burke–Ernzerhof (RPBE) functional, which incorporates the generalized gradient approximation method.^21,22^ Valence electrons received a detailed representation through Kohn–Sham wave functions, expanded within a plane-wave basis set,^23^ and were characterized with a cutoff parameter set at 520 eV.^24^ Meanwhile, core electrons were portrayed using a projector augmented-wave method. Rigorous standards were maintained to ensure the convergence of electronic energy to 10^−5^ eV and structural relaxation to 0.05 eV Å^−1^. The sampling of the Brillouin zone was executed using a Monkhorst–Pack grid, adhering to the criterion that the product of the number of *k*-points in any direction and the corresponding length of the basis vector, *k* × *a*, exceeds 15 Å. Spin-polarization was a critical aspect considered in all calculations. To facilitate adequate spacing, a vacuum layer of not less than 12 Å was established perpendicular to the surface. Lastly, the Atomic Simulation Environment (ASE) package was utilized for the efficient manipulation of crystal structures and the generation of inputs.^25^

We determined the electronic binding energies by employing the total energies of H_2_ and H_2_O as energy reference points, as delineated in the following eqn (1)–(3):

1 *E*_O*_ = *E*_slab-O*_ − *E*_slab_ − *E*_H_2_O_ + *E*_H_2__

2
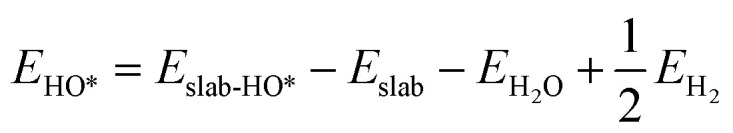
3
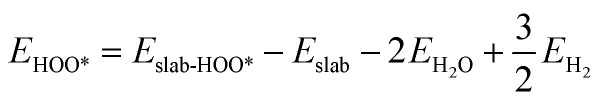
In these calculations, *E*_slab-ads*_ denotes the total energy of the surface with adsorbates attached, *E*_slab_ signifies the total energy of a bare slab surface, *E*_H_2_O_ represents the total energy of an H_2_O molecule in a vacuum, and *E*_H_2__ is the total energy of an H_2_ molecule also in a vacuum.

The binding free energies of all adsorbates on M–N–C catalysts were calculated using the computational hydrogen electrode (CHE) method.^26^ The calculation method includes the entropic, zero-point energy (ZPE), and solvation corrections, as shown in eqn (4). The results of these calculations are shown in Fig. S1.† For the adsorption-free energies of O_2_ and H_2_O_2_, we utilized the scaling relationship derived from previous studies,^27^ namely 
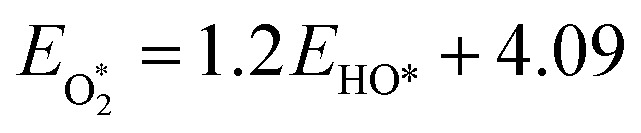
 and 
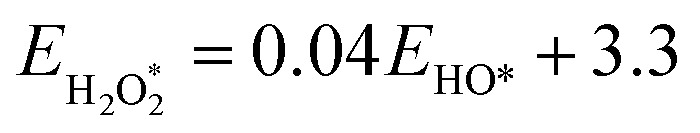
.

4 *G*_ads*_ = *E*_ads*_ + ΔZPE − *T*Δ*S* + *E*_solv_

### Electric fields and pH-dependent modelling at the RHE-scale

Electric fields were implemented through a saw-tooth potential corresponding to field strengths varying between −0.4 and 0.8 V Å^−1^ by employing the Quantum Espresso package,^28^ For each specified field, adsorbates underwent relaxation under a force convergence threshold set at 0.05 eV Å^−1^. The RPBE functional^21,22^ was used with an energy cutoff of 40 Ry and a density cutoff of 400 Ry. The gamma point was selected for all electric field computations. The lowest energy conformation from these relaxations was then utilized to predict the energy of the adsorbate under the respective electric field. The input file for Quantum Espresso is available in the Data availability section.

To describe the potential and pH dependence, we related the electric fields to both the standard hydrogen electrode (SHE) and reversible hydrogen electrode (RHE) potential using a parallel-plate capacitor model. The model is described by eqn (5), where *σ* refers to charge density, *ε*_0_ refers to the vacuum permittivity (8.85 × 10^−12^ F m^−1^), *ε* refers to the dielectric constant (unitless), *C*_H_ refers to the Helmholtz capacitance (μF cm^−2^), *U*_SHE_ refers to the potential *vs.* SHE, and *U*_PZC_ refers to the potential at the point of zero charges (PZCs) *vs.* SHE.5
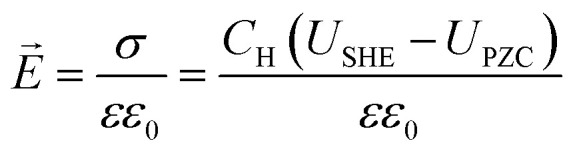


Fumagalli *et al.*^29^ demonstrated that the dielectric constant of water near a surface is two. The Helmholtz capacitance (*C*_H_) can vary with the surface and potential but typically ranges between 20 and 30 over the majority of the potential range, with more elevated values near the PZC. For simplicity, we assumed a constant *C*_H_ of 25 μF cm^−2^ across all surfaces.^14^

To gauge the response of an adsorbate to the applied electric field, we applied a second-order polynomial fitting to the calculation data for each adsorbate across the spectrum of field strengths. Subsequently, utilizing eqn (6), we determined the intrinsic dipole moment (*μ*) and polarizability (*α*) values for each adsorbate.6
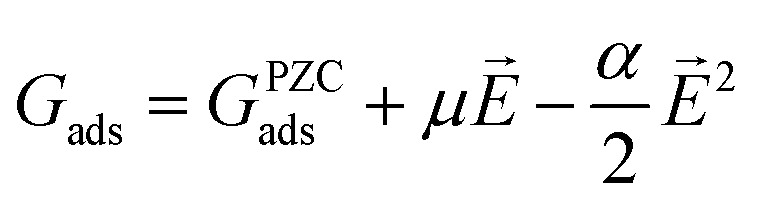
Herein, *G*^PZC^_ads_ refers to the binding energy of the adsorbate at the PZC, which corresponds to the energy calculated with no applied field. Our method for calculating binding energy dependence on the SHE potential differs from the methods employed in ref. 13 and 30. Our method requires the input of fixed PZC and *C*_H_, whereas their method allows PZC and *C*_H_ to vary with adsorption but relies on the ability of implicit solvent methods to accurately predict these values. However, the accuracy of these previous predictions has been called into question by other studies.^31,32^

The CHE was used to correct the binding energies for RHE dependence using eqn (7), where *n* refers to the number of electrons (relative to water), *e* refers to the charge of an electron, and *U*_RHE_ refers to the potential *versus* RHE.

7 *G*_ads_ = *G*_ads,*U*_RHE_=0_ − *neU*_RHE_

Ultimately, the free energy of an adsorbate at the given *U*_RHE_ and *U*_SHE_ is shown by eqn (8):8
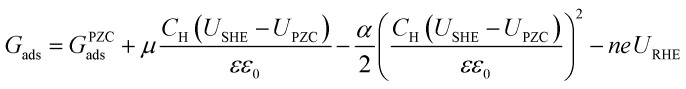


### Determining the PZCs

In our simulations, we configured the VASPsol parameters to their default settings. These included a bulk dielectric constant (*ε*_k_) of 78.4, a dielectric cavity width (*σ*) of 0.6, a cutoff charge density (*ρ*_cut_) of 0.0025 Å^−3^, and a surface tension parameter set at 0.525 meV Å^−2^. To facilitate accurate comparisons, our implicit model incorporated a graphene layer positioned beneath the catalysts. This addition aimed to neutralize the influence of lower-surface solvation on the slab. Trasatti *et al.*^33^ showed that *U*_PZC_ could be directly derived from the work function of a material in ion-free water *ϕ* using eqn (9):

9 *ϕ* = *eU*_PZC_ + *ϕ*_SHE_

where *ϕ*_SHE_ represents the absolute potential energy of the SHE. It is important to note that the value of *ϕ*_SHE_ can vary depending on the experiment conducted (ranging from 4.3 to 4.8 eV). However, in this study, the recommended value of 4.44 eV by the International Union of Pure and Applied Chemistry (IUPAC) was used.

### Microkinetic modeling of the ORR on M–N–C catalysts

The microkinetic modeling of the ORR volcano was based on our homemade codes with the approach pioneered by Hansen *et al.*^34^ and Kelly *et al.*^14^ along with the CatMAP package.^35^ Rates for intermediate steps were calculated using eqn (10):

10 Rate = *k*_f_Π*θ*_reac_ − *k*_r_Π*θ*_prod_

where *θ*_reac_ and *θ*_prod_ are the coverages of reactants and products, respectively. The rate constant *k* was calculated as the function of reaction perfector *A* (s^−1^), activation free energy *G*_a_, Boltzmann constant *k*_B_, and reaction temperature *T*:11
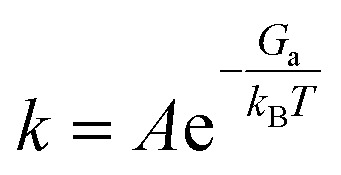


The intermediate reactions considered in the modeling are shown in reactions (12)–(19):

12 O_2_(aq) → O_2_(dl)

13
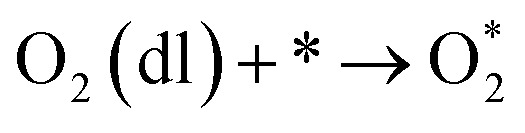
14
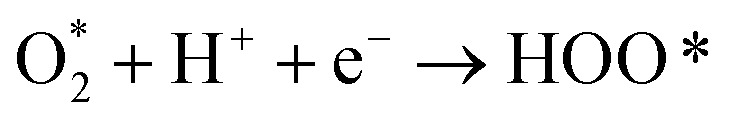

15 HOO* + H^+^ + e^−^ → O* + H_2_O(l)

16 O* + H^+^ + e^−^ → HO*

17 HO* + H^+^ + e^−^ → H_2_O(l) + *

18
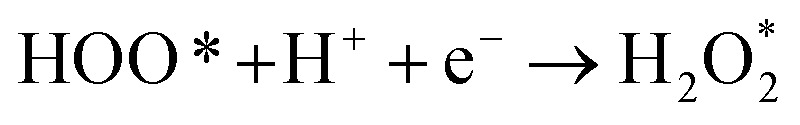
19
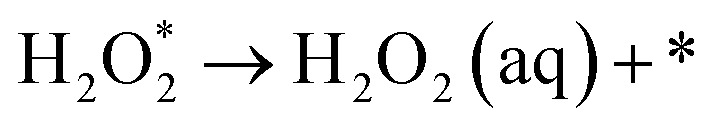


Reaction (12) describes the diffusion of aqueous O_2_ through a Nernstian diffusion layer, which can be generally modeled as a chemical step with a rate of 8 × 10^5^ s^−1^ (corresponding to an RDE rotation rate of 1600 rpm), as shown by Hansen *et al.*^34^ Reactions (14)–(18) involve proton–electron transfer steps, where the energy of the proton–electron pair is represented by the energy of half of an H_2_ molecule according to the CHE method.^26^ Reactions (14)–(18) describe the standard associative pathway for the 4e^−^ ORR, while reactions (17) and (18) represent the 2e^−^ ORR process. For reaction (15), where the O–O bond is broken along with protonation, we used eqn (20) developed by Dickens *et al.*^36^ to describe the activation energy of the O–O bond-breaking:

20 *G*_TS_ = 0.99*G*_HOO*_ − 0.25 + 0.42*U*_RHE_

For all other proton transfers, which did not include any other bond breaks, we used an intrinsic barrier of 0.26 eV and assumed 0.5 electrons had transferred at the transition state.^34^ Prefactors for all of the proton-electron transfer steps were set as 1 × 10^9^ s^−1^ to account for solvent reorganization.^14^

**Fig. 2:**
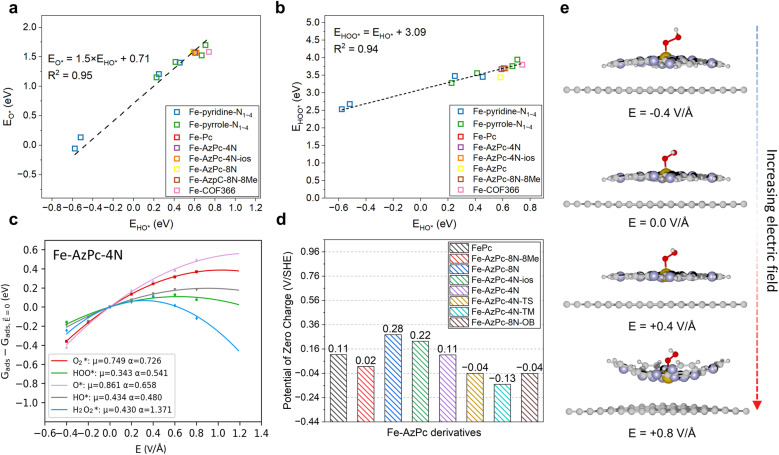
Factors for the pH-dependent microkinetic modeling of Fe–AzPc derivatives. Scaling relations between the binding energies of (a) HO* *vs.* O* and (b) HO* *vs.* HOO*. (c) Electric field effects on the adsorption energy of Fe–AzPc–4N. (d) Calculated potentials of zero charges (PZCs). (e) Under electric fields, the deformation of molecular M–N–C catalysts will lead to more significant field effects. Brown, gray, blue, red, and white spheres represent Fe, C, N, and H, respectively.

The energies used in the microkinetic modeling were adjusted using the scaling relations presented in Fig. 2 in the main text and were also corrected for the RHE potential and pH using eqn (7) and (8). The energies of aqueous and double-layer O_2_ were set to 5.19 eV at 0 V per RHE based on previous studies,^14,37^ and the O_2_ mole fraction was set to 2.34 × 10^−5^, corresponding to 1 atm O_2_ gas in equilibrium with water.

### Experimental methods

#### Catalyst preparation

30 mg of Ketjen Black (KB, EC300J, Lion Specialty Chemicals, Co. Ltd, Tokyo) was dispersed in a dimethyl sulfoxide (DMSO) solution of 0.1 mg mL^−1^ metal–AzPc. The dispersion was sonicated with a homogenizer for 5 min and then suction-filtered to collect the samples. The samples were washed three times each with methanol and chloroform and then dried *in vacuo*. Details of the experimental synthesis and characterization studies are available in the ESI.†

## Results and discussion

First, we identified a reliable reaction descriptor for the ORR over Fe-based M–N–C catalysts by linear scaling relation analysis on the ORR adsorbates (O*, HO*, and HOO*) across various Fe-based M–N–C catalysts (Fig. 2a and b), including classic Fe–pyridine–N_*x*_, Fe–pyrrole–N_*x*_, FePc, Fe–COF366,^10^ and Fe–AzPcs (Fig. 1). Both the relationships of HO* *vs.* O* and HO* *vs.* HOO* follow good linear trends with high *R*^2^ values (0.95 and 0.94, respectively), showing that HO* binding energy can be a good reaction descriptor along with the regression equations displayed in Fig. 2a and b. Interestingly, for the scaling relation of *E*_HO*_vs. E_O*_, the intercept is ∼0.71 with a slope of 1.5, which is higher than that of transition metals.^38^ This is because of the atop-site adsorption of O* and HO*, leading to relatively weaker O* bonding at a given HO* bonding strength due to the stronger repulsive interaction between O* and metal-atop.^39^ In terms of the scaling relation of *E*_HO*_vs. E_HOO*_, the intercept is ∼3.09 with a slope of 1, which fits into the well-known universal scaling relation of *E*_HO*_vs. E_HOO*_ (*E*_HOO*_ = *E*_HO*_ + 3.2 ± 0.2 eV).^40^ Next, to model the pH effect on the reaction energetics, electric field effects on the adsorbate binding energies are the key information to derive the intrinsic dipole moment change (*μ*) and polarizability (*α*) based on fitting the field-induced adsorption energy change across various applied fields, using a second polynomial model (Fig. 2c).^14^ Note that a classic method, which treats the pH-dependent energetic correction as a consistent term (*i.e*., 0.059 × pH), fails to predict the pH-dependent ORR performance on an RHE-scale because the SHE-scale shifts with the same constant (0.059 × pH) as a function of pH. From the electric field simulation, it can be seen that the dipole moment (*μ*) and polarizability (*α*) upon adsorption are generally larger than those of transition metals.^14^ This is also due to the atop-site adsorption of ORR adsorbates over these catalysts, leading to a larger dipole moment and polarizability than those of the hollow- or bridge-site adsorption on transition metal surfaces.^3^ To model the pH effect, PZC is another key factor.^14^ Recent studies have found that for a catalyst that does not lead to significant water oxidation at the potential of interest, PZCs acquired from an implicit solvent can lead to good agreement with experiments.^41^ Interestingly, Fig. 2d shows that while other Fe-based M–N–Cs generally show positive PZC values, the three more stereoscopic Fe–AzPcs (with a “dancing pattern”) have negative/close-to-zero PZCs (−0.04, −0.13, and −0.04 V_SHE_ for Fe–AzPc–4N–TS, Fe–AzPc–4N–TM, and Fe–AzPc–8N–OB, respectively). To guarantee the modeling accuracy, all the scaling relations and effects analyzed in Fig. 2 will be included in the pH-dependent microkinetic modeling at an RHE scale, which will be discussed later in this paper. Note that in general, a more positive field corresponds to a lower pH, and the energetic changes under various fields are related to the field-induced structural relaxation in different degrees (Fig. 2e). Interestingly, we observed a significant deformation (with a larger curvature) of these catalysts under a high electric field. Details of all the computation and modeling methods can be found in the Methods section.

**Fig. 3:**
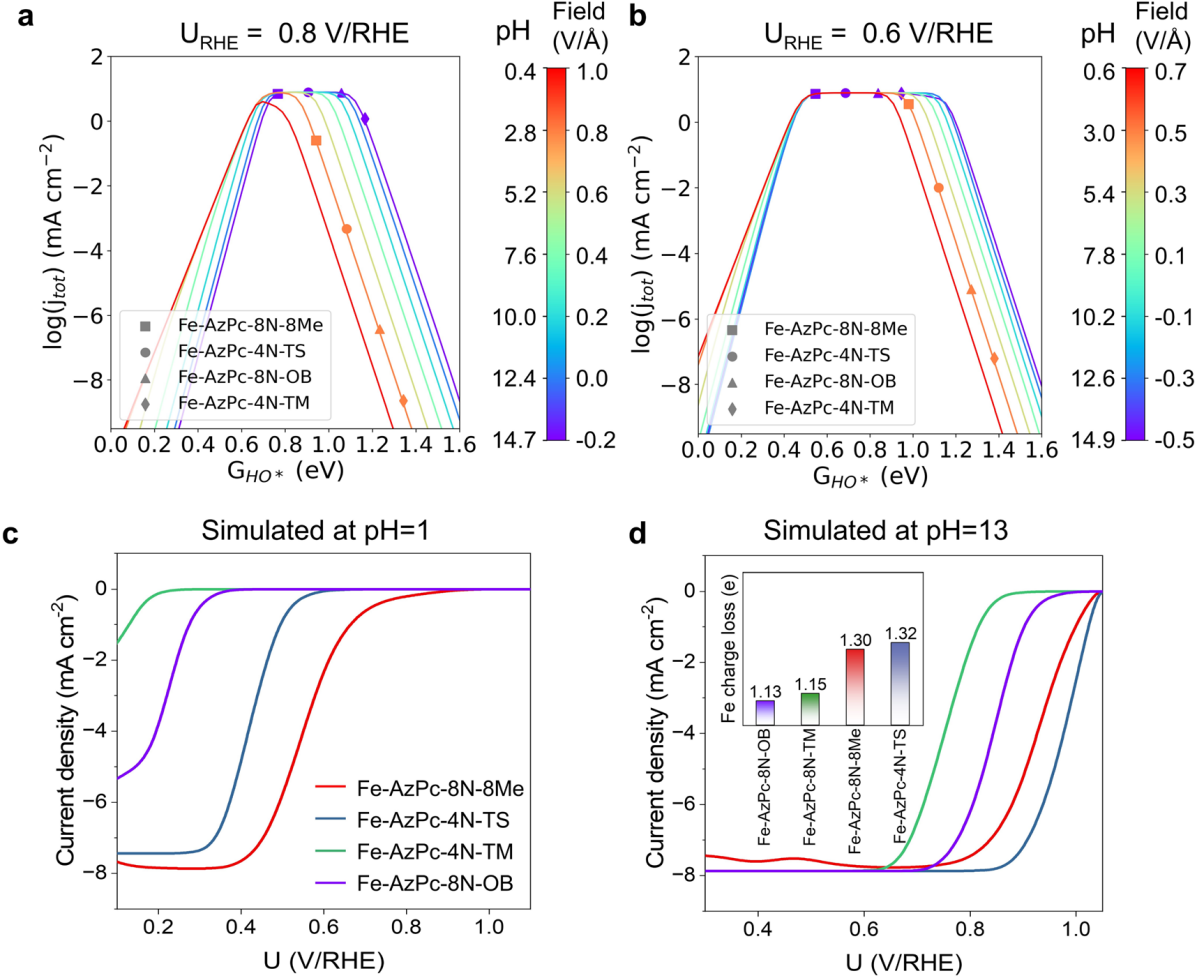
pH-dependent ORR volcano models and the simulated LSV curves of Fe–AzPc derivatives. pH-field dependent volcanos simulated at (a) 0.8 V per RHE and (b) 0.6 V per RHE, as a function of *G*_HO*_. The left and right sides of the color bar represent the correlation between the electric field and pH, respectively, with the PZCs adopting the average value from Fig. 2d, which is 0.07 V per SHE. Simulated LSV curves at (c) pH = 13 and (d) pH = 1. The microkinetic modeling considered all the acquired parameters and factors from Fig. 2.

With the details of scaling relations as a function of HO* binding energy, dipole moment and polarizability upon adsorption, and the calculated PZCs (Fig. 2), we performed pH-dependent microkinetic modeling by considering the derived kinetics and thermodynamics of possible elementary steps in the ORR (Fig. 3; details of the microkinetic modeling can be found in the Methods section). First, we derived the pH-dependent volcano activity model as a function of potential (*i.e*., 0.8 and 0.6 V per RHE) and the binding free energy of HO* (*G*_HO*_) (Fig. 3a and b). Interestingly, it can be seen in Fig. 3a (at a potential of 0.8 V per RHE) that under more acidic conditions, there is a slight left-and-down shift of the volcano, with the predicted maximum current density lower than that under more alkaline conditions and a more positive optimal *G*_HO*_. At a potential of 0.6 V per RHE, the maximum current density is the same regardless of the pH (Fig. 3b); but still, there is a slight left-shift of the volcano under more acidic conditions. All these originated from the large dipole moment and polarizability change upon adsorption. Note that on close-packed transition metal surfaces, this pH-dependent volcano-shift is not significant at 0.8 V per RHE,^14^ suggesting that M–N–C catalysts are a class of catalysts with distinct ORR behaviors. Next, we simulated the ORR linear sweep voltammetry (LSV) curves at pH = 13 (Fig. 3c) and pH = 1 (Fig. 3d) for comparison with the subsequent experiments, by considering each Fe–AzPc's energetics, dipole moments, polarizabilities, and PZCs (Fig. 2c and d). According to the simulated current density results, we found that both Fe–AzPc–8N–Me and Fe–AzPc–4N–TS exhibit relatively good ORR activity under acidic and alkaline conditions, while Fe–AzPc–8N–OB and Fe–AzPc–4N–TM show comparatively poor performance. Additionally, we conducted a Bader charge analysis on the four structures (Fig. 3d, inset); the results indicate that the Fe atoms in Fe–AzPc–8N–Me and Fe–AzPc–4N–TS lose about 0.2 more electrons than those in Fe–AzPc–8N–OB and Fe–AzPc–4N–TM, with a total electron loss of 1.3 electrons. The charge transfer could be due to both the influence of the long-chain functional groups present in the AzPc catalysts and the bending or twisting of the graphene. Given that all AzPc derivative catalysts are distributed on graphene surfaces, we suggest that the long-chain functional groups are more significant in facilitating the transfer of electrons. Therefore, in Fe–AzPcs with long-chain functional groups, there is a clear correlation between the number of electrons lost and the catalytic performance of the Fe atom.

**Fig. 4:**
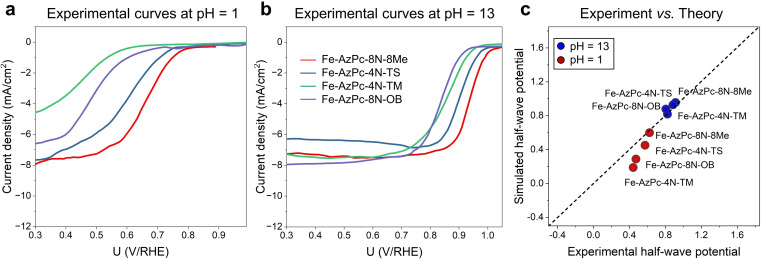
Experimental RDE polarization curves are provided at (a) pH = 1 and (b) pH = 13. (c) Direct comparison between the experimental and simulated half-wave potentials.

To provide a direct comparison with our pH-field-dependent simulations above, we conducted ORR experiments on the above four typical Fe–AzPc structures (Fig. 4). Herein, we followed the mature synthetic methods from previous studies.^18–20^ Details of the synthetic, characterization, and electrochemical test methods are shown in the ESI,† and other evidence of the catalysts' structural information can be found in ref. 18–20. Excitingly, the experimental rotating disk electrode (RDE) curves at a pH of 1 (Fig. 4a) and 13 (Fig. 4b) show trendily good agreements with our theoretical simulations (Fig. 3), with the pH-dependent performance trends highly consistent with the simulated performances on these Fe–AzPcs by considering the key effects on the ORR. Under alkaline conditions, the half-wave potentials observed in Fe–AzPcs generally exceed those of Pt/C catalysts, as shown by the polarization curves of Pt/C presented in Fig. S15.† Under acidic conditions, the theoretical prediction of the half-wave potential for the Fe–AzPc-based catalysts is slightly lower than the experimental values. This discrepancy can be attributed to two main factors: the first is the possible difference in the density of reactive sites in the model compared to the actual catalyst. When the reactivity of these sites is sufficiently high, such as under alkaline conditions, the reaction current density depends on the oxygen molecules entering the double-layer interface rather than on the density of reactive sites. However, when the intrinsic activity of the reactive sites is relatively low, the difference between the experimental and theoretical reactive sites can cause a deviation in the predicted values of the theoretical model. The second reason is that the computational hydrogen electrode (CHE) model typically assumes that the elementary steps of the electrochemical reaction occur at a constant electric field rather than a constant electrode. Therefore, the PZC change during the electrochemical reaction may also affect the predicted half-wave potentials. Although these two factors may affect the quantitative comparison between experiment and theory, they do not impact the theoretical prediction of the relative performance of different long-chain functional groups. Therefore, this model remains highly effective in screening for long-chain surrounding functional groups with superior performance of Fe–AzPcs for the ORR. The stability of AzPc catalysts is crucial for their real-world application. We have evaluated the durability of Fe–AzPc–8N–8Me and Fe–AzPc–4N–TS, in comparison with Fe–Pc (acquired from Tokyo Chemical Industry Co., Ltd, Japan) and Pt/C. Our results reveal that, in an alkaline environment, the stability of Fe–AzPc–8N–8Me significantly surpasses that of FePc and Pt/C (Fig. S16a†). Furthermore, the durability of Fe–AzPc–8N–8Me was assessed through its application in a Zn–air battery (Fig. S16b†), with comprehensive details available in our previously published paper.^19^ The discharge experiments demonstrated that the battery could sustain over 10 hours of operation, with the potential decrease predominantly attributed to the degradation of the anode Zn foil. These results affirm the high durability of AzPc catalysts, underscoring their suitability for practical battery cell applications.

## Conclusion

In this benchmarking study, we have performed comprehensive microkinetic modeling and compared the results with experimental observations using the emerging large-scale Fe–AzPc catalysts. We have conducted pH-dependent microkinetic modeling analysis based upon *ab initio* calculations to analyze the pH-dependent ORR performance of four typical carbon-supported Fe–AzPcs with varying long-chain functional groups and compared these results with those of subsequent ORR experiments. We found that although all these Fe–AzPcs may have the same active center (*i.e*., the Fe-site), different “dancing patterns” lead to very different ORR performances due to the different surrounding functional groups, which is attributed to the change of the electronics of the Fe-site induced by different degrees of the structural relaxation of a molecule. We also found that the charge loss number of the central atom Fe can serve as an approximate descriptor for the ORR. Combined with the pH-dependent volcano plot developed in this work, it enables rapid prediction of ORR performance without the need for adsorption-induced geometric optimization of each long-chain AzPc. These long-chain AzPcs, including carbon-based substrates, typically have over 650 atoms, making their optimization extremely computationally intensive. Our results show that the pH-field coupled microkinetic modeling method for the ORR can lead to good agreement with experimental observations at different pH, which can provide precise predictive insights into the ORR activities of M–N–C catalysts. This study also suggested that in the catalytic design of molecular M–N–C materials, the aspect of pH-dependency warrants careful consideration.

## Data availability

The data that support the findings of this study are available from the corresponding authors upon reasonable request. Other information, *e.g*., the relaxed structures of the large molecule Fe–AzPcs, can be found *via* the link https://github.com/M-N-Cs/Fe-AzPcs.

## Author contributions

H. Li and H. Yabu conceived the idea and supervised this work. D. Zhang, Y. Hashimoto, H. Yabu and H. Li wrote the manuscript. D. Zhang performed DFT calculations and pH-field coupled microkinetic modelling. Y. Hirai, K. Nakamura, and K. Ito prepared the Fe azaphthalocyanine catalysts and tested their performance and durability. Y. Matsuo and K. Ishibashi conducted the structural characterizations for the catalysts. All authors discussed and analyzed the results during manuscript preparation.

## Conflicts of interest

There are no conflicts to declare.

## Supplementary Material

SC-015-D4SC00473F-s001
