# Supplementary material for: Benchmarking pH-field coupled microkinetic modeling against oxygen reduction in large-scale Fe–azaphthalocyanine catalysts
Source: Chem Sci. 2024 Mar 15;15(14):5123–32. doi: 10.1039/d4sc00473f (PMC10988579; doi:10.1039/d4sc00473f)
Supplement: SC-015-D4SC00473F-s001 [file SC-015-D4SC00473F-s001.pdf]

Electronic Supplementary Material (ESI) for Chemical Science.
This journal is © The Royal Society of Chemistry 2024

Electronic Supplementary Information

Benchmarking pH-Field Coupled Microkinetic Modeling Against Oxygen Reduction in Large-Scale Fe-Azaphthalocyanine Catalysts

Di Zhang[1], Yutaro Hirai[2], Koki Nakamura[2], Koju Ito[2], Yasutaka Matsuo[3], Kosuke Ishibashi[1], Yusuke Hashimoto[4], Hiroshi Yabu[1,*], and Hao Li[1,*]

[1] Advanced Institute for Materials Research (WPI-AIMR), Tohoku University, Sendai 980-0811, Japan

[2] AZUL Energy, Inc., 1-9-1, Ichibancho, Aoba-Ku, Sendai 980-0811, Japan

[3] Research Institute for Electronic Science (RIES), Hokkaido University, N21W10, Sapporo 001-0021, Japan

[4] Tohoku Forum for Creativity, Tohoku University, Sendai 980-8577, Japan

*** Corresponding Authors:**

Email: hiroshi.yabu.d5@tohoku.ac.jp (H. Y.)

Email: li.hao.b8@tohoku.ac.jp (H. L.)

1. Computational Methods

Supplementary Table 1. Summary of the DFT-calculated energies, experimental Gibbs free formation energies, and entropic contributions at standard conditions: T=298 K and pressure=1 bar. Unit: eV

Species	E^{DFT}	ZPE	$T\Delta S^{exp}$	$ZPE - T\Delta S$	E_{solv}
H_2O (l)	−14.1476	0.57	0.67 (0.035 bar)	−0.10	-
H_2 (g)	−6.9897	0.35	0.41	−0.06	-
O*	-	0.066	0	0.066	-
HO*	-	0.343	0	0.343	−0.15
HOO*	-	0.403	0	0.403	−0.40
O_2*	-	0.135	0	0.135	-

2. Chemical Structures without Long Stretching Functional Groups

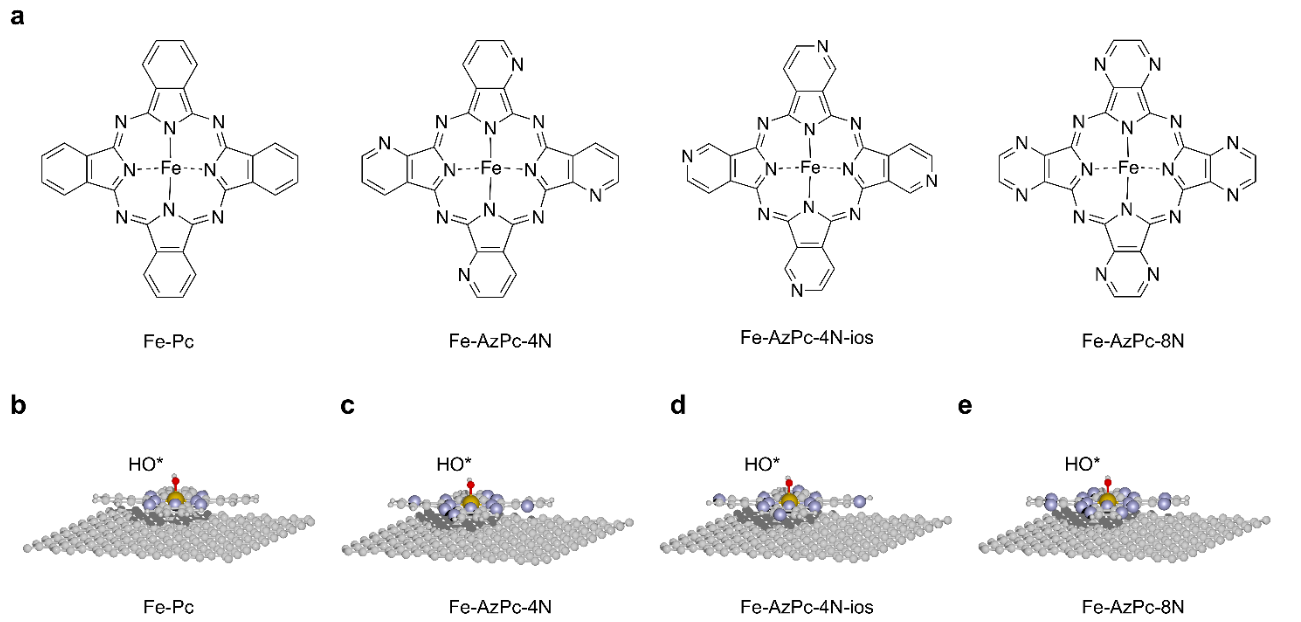

Figure S1. Structures of Fe-Azaphthalocyanines (AzPc) molecular catalysts without long-chain functional groups. (a) Chemical structures of Fe-Pc, Fe-AzPc-4N, Fe-AzPc-4N-ios, and Fe-AzPc-8N. After DFT geometric relaxations, these structures are shown in (b) Fe-Pc, (c) Fe-AzPc-4N, (d) Fe-AzPc-4N-ios, and (e) Fe-AzPc-8N, resembling molecules dancing on the 'stage' of graphene. Yellow, gray, blue, red, and white spheres represent Fe, C, N, O, and H, respectively.

3. Synthesis and characterization of catalyst molecules

3.1 Common procedures

Matrix assisted laser deposition / ionization time of flight mass spectroscopy (MALDI-TOF-MS) measurement of catalytic molecules were performed using REFLEXIII, Bruker Daltonics with alpha-Cyano-4-hydroxycinnamic acid (CHCA) as a matrix. UV-Vis spectra of catalyst molecules dissolved in DMSO (WAKO/GR) have been measured by using UV-Vis-NIR spectrometer, V-670, Jasco, Tokyo, Japan. Each sample was dispersed in DMSO by ultrasonication for 10 min and the spectra of supernatants, whose wavelength ranging from 300 to 800 nm, were measured.

3.2 Respective preparation methods

1) **FeAzPc-8N-8Me** ((*SP*-4-1)-[2,3,9,10,16,17,23,24-Octamethyl-29*H*,31*H*-tetrapyrazino[2,3-*b*:2′,3′-*g*:2″,3″-*l*:2‴,3‴-*q*]porphyrazinato(2-)-κN^{29},κN^{30},κN^{31},κN^{32}]iron, CAS:1638644-65-1)

Synthetic route of FeAzPc-8N-8Me is shown in **Figure S2**. The detailed procedure for preparation is outlined in the following steps:

Synthesis of 5,6-dimethyl-2,3-pyrazinedicarbonitrile: 60 mL of acetone and 1 mL of acetic acid were slowly mixed with 10.8 g (100 mmoL) of diaminomaleonitrile (100 mmoL) in a three-neck flask with stirring. 8.6 g (100 mmoL) of diacetyl was added dropwise to the solution. After mixing, the mixture was heated and refluxed gently for 1 hour, then 150 mg of activated carbon was added and stirred for 30 minutes. The mixture was filtered with heating through Celite filtration to remove insoluble material. The filtrate was concentrated by evaporation, and isopropanol was added to the residue and stirred. The precipitated crystals were filtered off, washed with cold isopropanol, and dried under reduced pressure to obtain the desired product. Yield: 14.7 g (92.9 %).

Synthesis of FeAzPc-8N-8Me: 19.8 g of iron(II) chloride tetrahydrate was added to 300 mL of 1-pentanol with stirring. By heating, 100 mL of 1-pentanol was distilled off under normal pressure. The mixture was allowed to cool to 130 °C, and 60.1 g of 5,6-dimethyl-2,3-pyrazinedicarbonitrile was added. The temperature was set to 155 °C and the reaction was carried out for 15 hours. The reaction

mixture was cooled to 100 °C, 100 mL of 6N hydrochloric acid was added, and stirred for 2 hours at 100 °C. The mixture was cooled to 70 °C, and the precipitated solid was filtered off. After washing with methanol, the solid was washed with 2N hydrochloric acid, followed by methanol, and finally acetone.

The product was dried at 60 °C under reduced pressure to obtain the desired product. Yield: 49.2 g (75.2 %).

Characterization: The structure of yielded compound was confirmed by MALDI-TOF-MS spectrum (**Figure S3**, left side plot). UV-Vis spectrum of its DMSO solution are shown **in Figure S5**, right side plot. MALDI-TOF-MS: Calculation: 688.501, Measured: m/z=689.440, UV-Vis: λ_{max}= 626 nm

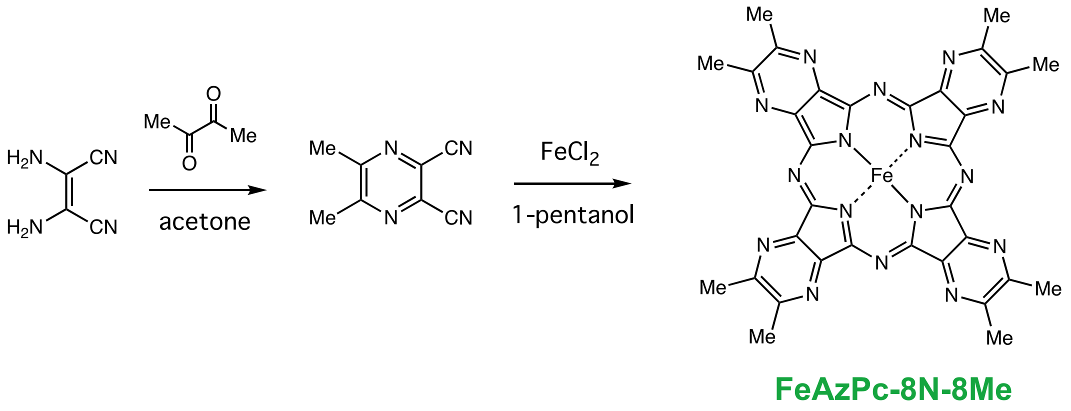

Figure S2. Synthetic route of FeAzPc-8N-8Me.

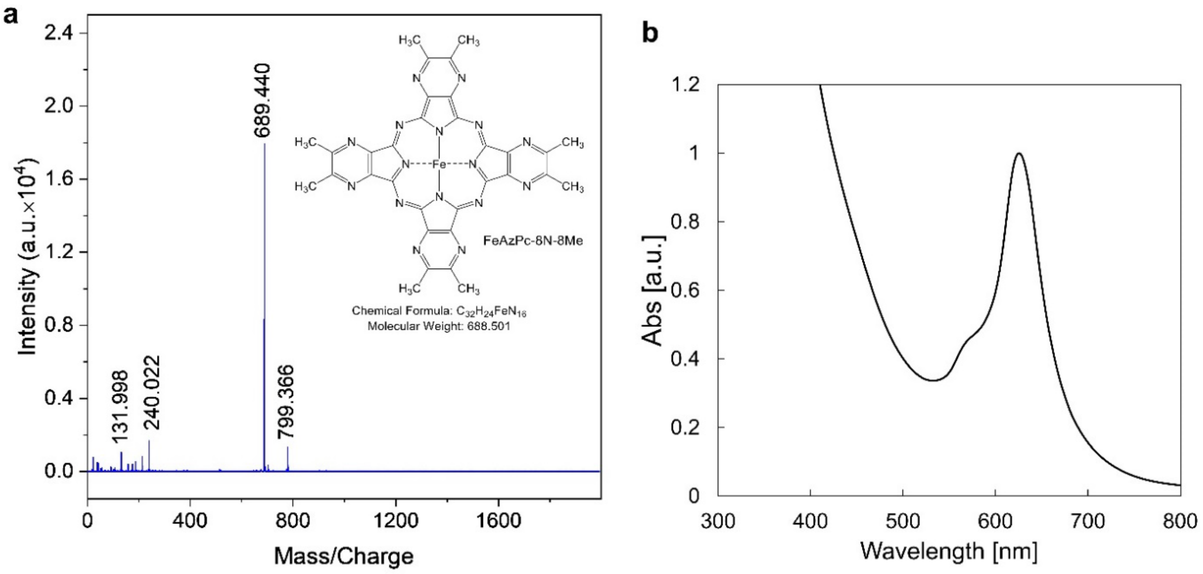

Figure S3. MALDI-TOF-MS spectrum of FeAzPc-8N-8Me (**a**) and UV-Vis spectrum of FeAzPc-8N-8Me/DMSO solution (**b**), respectively.

2) **FeAzPc-4N-TS** ((*SP*-5-12)-Chloro[3,10,17,24-tetrakis[(2-ethylhexyl)sulfonyl]-29*H*,31*H*-tetrapyrido[2,3-*b*:2′,3′-*g*:2″,3″-*l*:2‴,3‴-*q*]porphyrazinato(2-)-κN^{29},κN^{30},κN^{31},κN^{32}]iron, CAS:2793446-29-2)

Synthetic route of FeAzPc-4N-TS is shown in **Figure S4**. The detailed procedure for preparation is outlined in the following steps:

Synthesis of 5-Bromo-2,3-pyridinedicarboxylic acid dimethyl ester: 30 g quinolinic acid (179.5 mmoL) was added to 250 mL methanol and stirred. 10 mL of concentrated sulfuric acid was added dropwise, followed by the addition of 20 mL of methyl orthoformate and refluxed. After one day refluxing, 20 mL of methyl orthoformate was added, and the solution was refluxed overnight. The solution was cooled to 40 °C, and 16 mL of bromine was added dropwise, and after one day, another 8 mL of bromine was added, and the bath temperature was set at 58 °C. The reaction was carried out for another two days. After cooling, 300 mL of ethyl acetate and 150 mL of water were added, and the solution was neutralized with sodium hydrogen carbonate, and after separation, the organic phase was washed twice with saturated sodium hydrogen carbonate solution. The organic phase was dehydrated with anhydrous sodium sulfate, concentrated by evaporation, and the product was crystallized with cold isopropyl alcohol. The resulting crystals were filtered out, washed with cold isopropyl alcohol, and dried to obtain the product. Yield: 34.4 g (69.9 %)

Synthesis of 5-(2-ethylhexylsulfanyl)-2,3-pyridinedicarboxylic acid dimethyl ester: 10.0 g 2-ethylhexanethiol (68.4 mmoL), 18.7 g dimethyl 5-bromopyridine-2,3-dicarboxydimethyl (68.2 mmoL), and 14.7 g potassium carbonate were added to 80 mL DMF, the solution was heated at 80 ˚C under N_2 atmosphere overnight. After reaction, the solvent was distilled off under reduced pressure. Water and ethyl acetate were added, and the organic phase was separated and washed twice with dilute hydrochloric acid, followed by washing with water, dehydration with anhydrous sodium sulfate, and concentrated under reduced pressure to obtain the product. Yield: 20.0 g (86.1%).

Synthesis of 5-(2-ethylhexylsulfonyl)-2,3-pyridinedicarboxylic acid dimethyl ester: 120 mL acetic

acid and 780 mg sodium tungstate, dihydrate were added to 15.5 g 5-(2-ethylhexylsulfonyl)-2,3-pyridinedicarboxylic acid dimethyl ester (45.7 mmoL) and heated to 40 ˚C. A half of 11.4 mL of 30% hydrogen peroxide aqueous solution was added dropwise to the solution, and the mixture was stirred for 30 minutes. After the temperature was set to 75 °C, the remaining hydrogen peroxide aqueous solution was slowly added dropwise. The reaction was carried out at 75 °C for 2 hours and then, cooled to room temperature. Ethyl acetate and water were added, and organic phase was separated from the mixture. The organic phase was washed three times with sodium sulfite solution, followed by three washes with saturated sodium bicarbonate solution, and the organic phase was dehydrated with anhydrous sodium sulfate. The organic phase was concentrated, and the residue was purified by silica gel column chromatography to give the product as a colorless oil. Yield: 14.5 g (85.4 %).

Synthesis of 5-(2-ethylhexylsulfonyl)-2,3-pyridinedicarboxylic acid: 100 mL of methanol was added to 6.0 g (16.2 mmoL) of 5-(2-ethylhexylsulfonyl)-2,3-pyridinedicarboxylic acid and stirred at room temperature. 3.0 g of lithium hydroxide monohydrate was added to this solution, and the reaction was carried out at room temperature for 1 hour. After confirming the end of the reaction by TLC, the solution was acidified with concentrated hydrochloric acid and the solvent was removed under reduced pressure. The residue was extracted with ethyl acetate, washed three times with water, and the organic phase was concentrated to give a colorless oil-like product, which solidified after standing overnight. Yield: 5.0 g (89.9 %).

Synthesis of FeAzPc-4N-TS: 3.0 g of urea and 25.3 mg of ammonium molybdate, hexahydrate was added to 1.0 g of 5-(2-ethylhexylsulfonyl)-2,3-pyridinedicarboxylic acid, and the mixture was stirred at 160 °C for 30 minutes under nitrogen flow. 400 mg of iron(III) chloride, hexahydrate, and 3.0 g of urea were added to this reaction mixture, and the reaction was carried out at 200 °C for 3 hours. After cooling to room temperature, water was added and the precipitated solid was filtered off, washed with water, washed with methanol, and dried to give the crude product. This was purified by silica gel column chromatography to obtain the desired product. Yield: 201 mg (21.6%).

Characterization: The structure of yielded compound was confirmed by MALDI-TOF-MS spectrum (**Figure S5**, left side plot). UV-Vis spectrum of its DMSO solution were shown in **Figure S5**, right side plot. MALDI-TOF-MS: Calculation: 1,277.429, Measured: m/z=1279.736 (including some fragments), UV-Vis: λ_{max}= 653 nm.

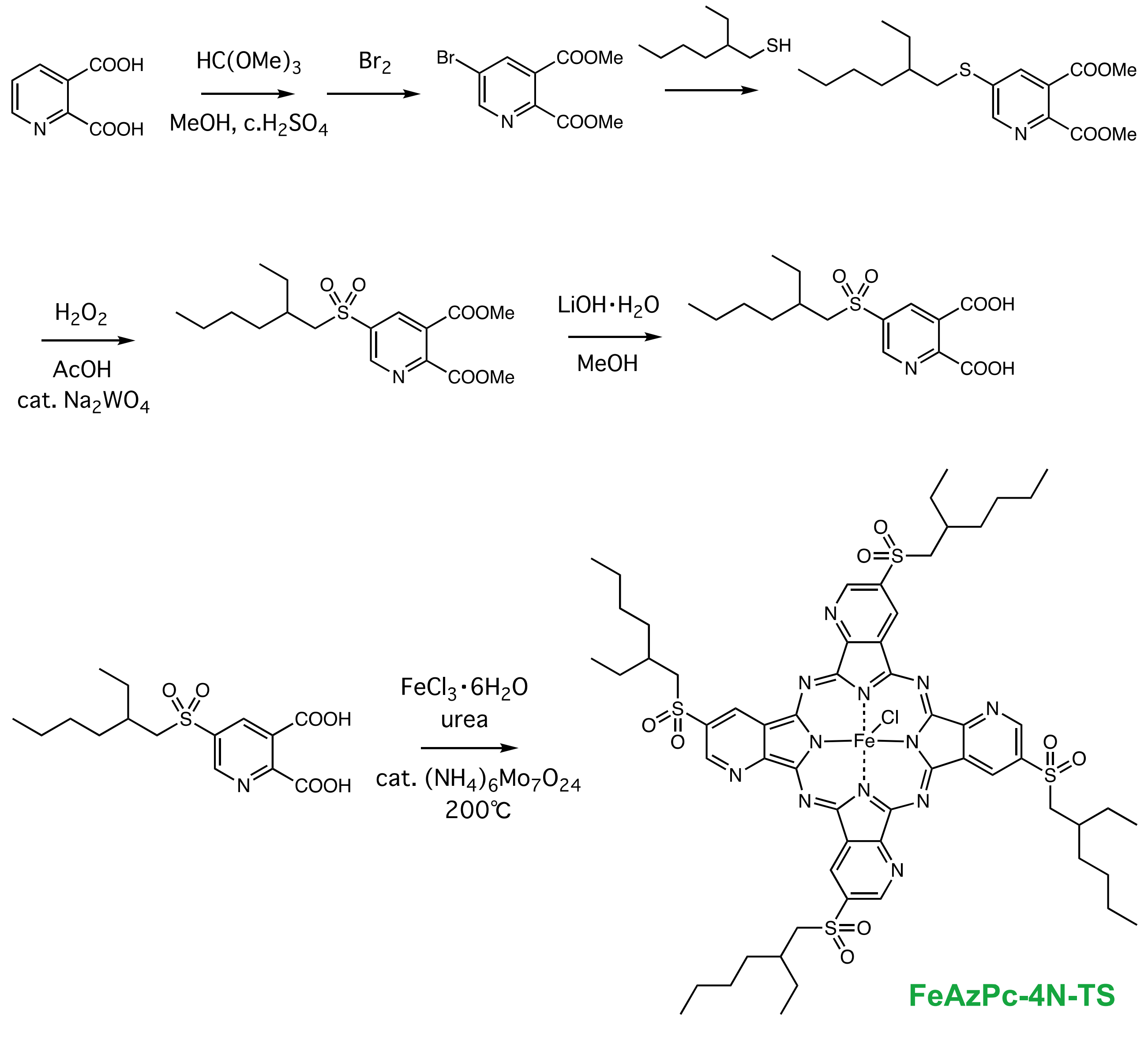

Figure S4. Synthetic route of FeAzPc-4N-TS.

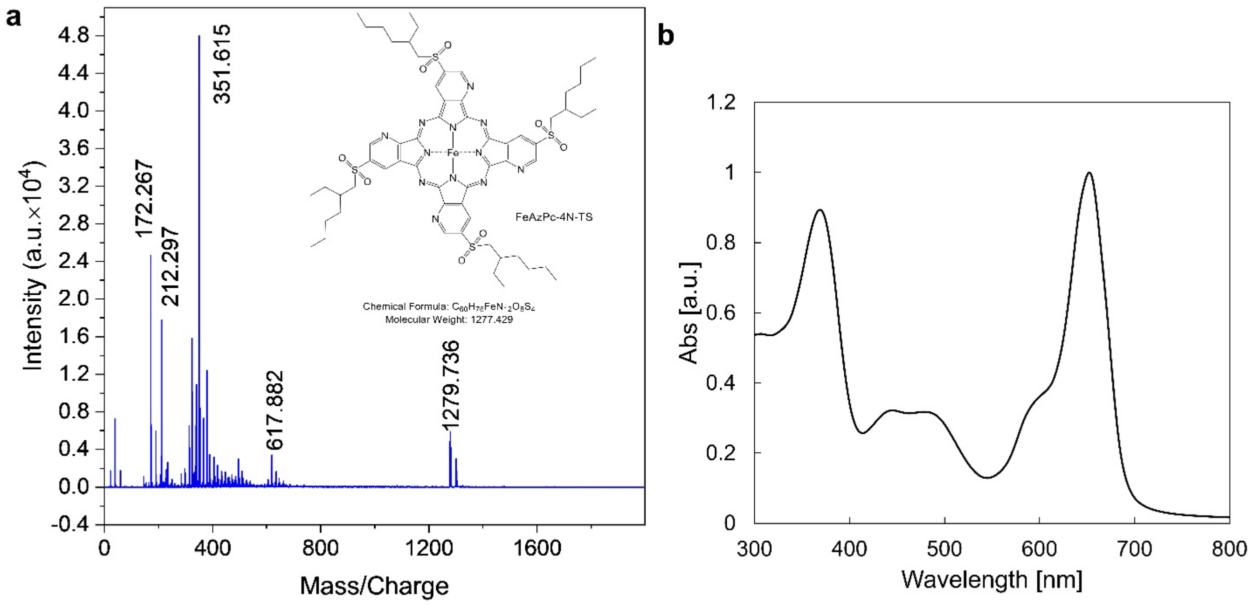

Figure S5. MALDI-TOF-MS spectrum of FeAzPc-4N-TS (**a**) and UV-Vis spectrum of FeAzPc-4N-TS /DMSO solution (**b**), respectively.

3) **FeAzPc-4N-TM** ((*SP*-5-12)-Chloro[3,10,17,24-tetrakis[(2-ethylhexyl)thio]-29*H*,31*H*-tetrapyrido[2,3-*b*:2′,3′-*g*:2″,3″-*l*:2‴,3‴-*q*]porphyrazinato(2-)-κN^{29},κN^{30},κN^{31},κN^{32}]iron, CAS:2793446-28-1)

Synthetic route of FeAzPc-4N-TS is shown in **Figure S6**. The detailed procedure for preparation is outlined in the following steps:

Synthesis of 5-(2-ethylhexylsulfanyl)-2,3-pyridinedicarboxylic acid: 80 mL of methanol was added to 5.0 g of 5-(2-ethylhexylsulfanyl)-2,3-pyridinedicarboxylic acid, which was synthesized in the process of FeAzPc-4N-TS, and stirred. Then 2.8 g of lithium hydroxide monohydrate was added and stirred overnight at room temperature. The mixture was acidified with dilute hydrochloric acid, and water and ethyl acetate were added for separation. The organic phase was washed with water three times and concentrated under reduced pressure until it dried, desired object was obtained. Yield: 4.2 g (91.6 %).

Synthesis of FeAzPc-4N-TM: 6.3 g of urea and 53.1 mg of ammonium molybdate, tetrahydrate were added to 1.9 g of 5-(2-ethylhexylsulfanyl)-2,3-pyridinedicarboxylic acid and the mixture was stirred at 160 °C for 30 minutes under nitrogen flow. 840 mg of iron(III) chloride, hexahydrate, and 6.3 g of urea were added to the reaction mixture and the reaction was carried out at 195 °C for 5 h. After cooling to 100 °C, 100 mL of water was added, and the mixture was stirred overnight. The precipitated solid was filtered out, washed with water, washed with methanol, and purified by silica gel column chromatography. Yield: 858 mg (48.9 %).

Characterization: The structure of yielded compound was confirmed by MALDI-TOF-MS spectrum (**Figure S7**, left side plot). UV-Vis spectrum of its DMSO solution are shown in **Figure S8**, right side plot. MALDI-TOF-MS: Calculation: 1,149.437, Measured: m/z=1148.986 (including some fragments), UV-Vis: λ_{max}= 650 nm

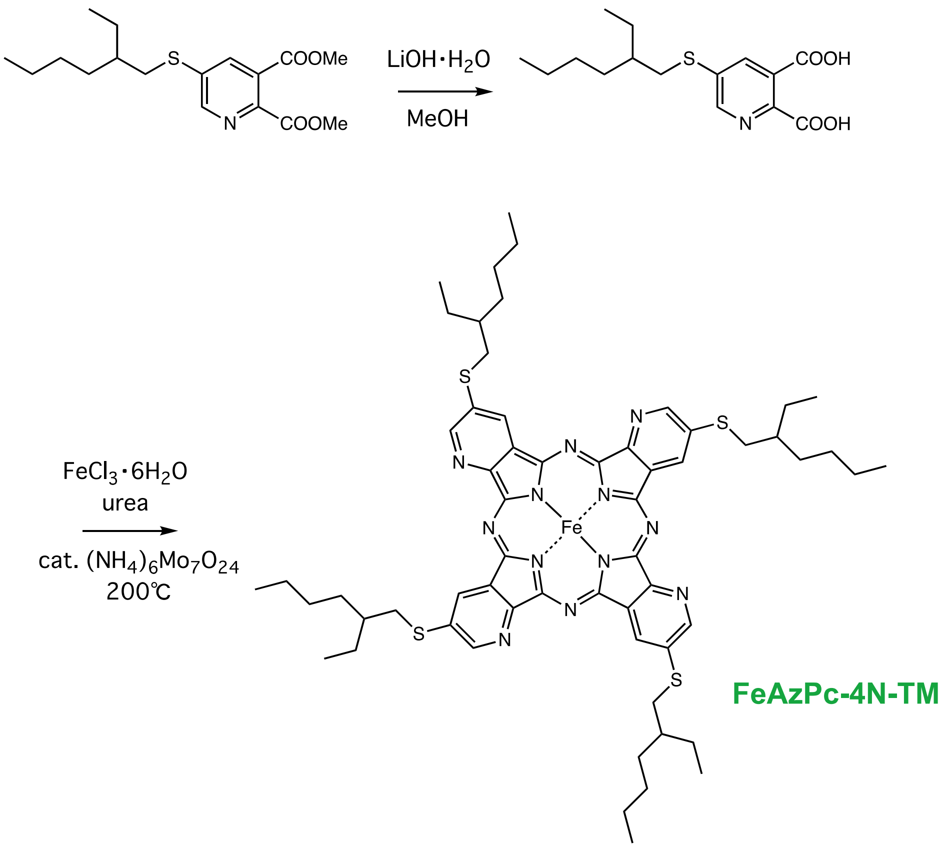

Figure S6. Synthetic route of FeAzPc-4N-TM.

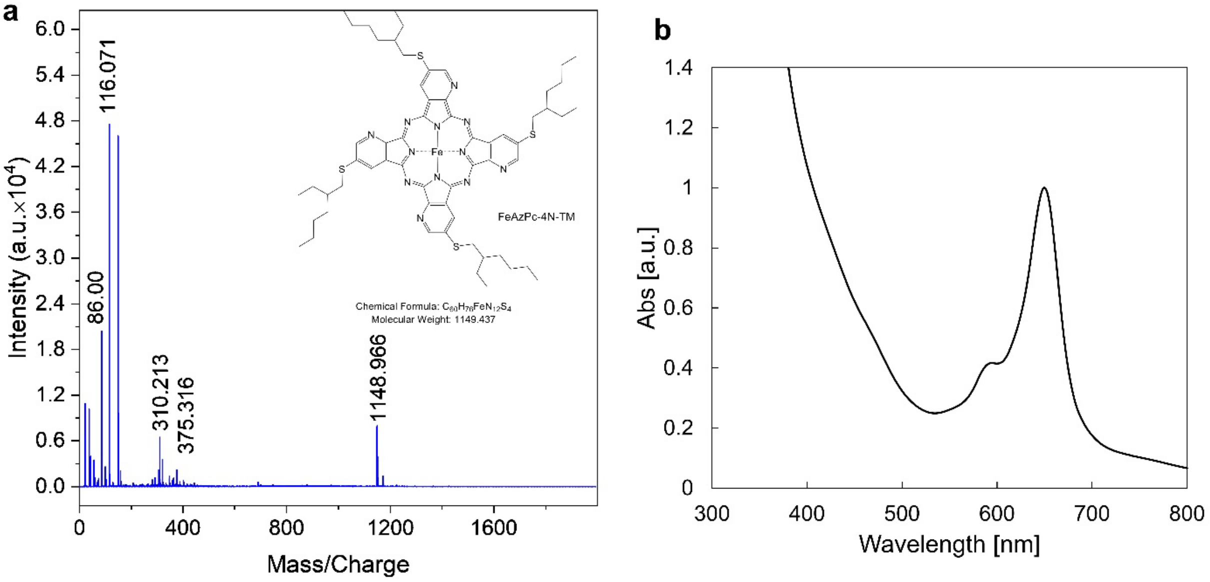

Figure S7. MALDI-TOF-MS spectrum of FeAzPc-4N-TM (**a**) and UV-Vis spectrum of FeAzPc-4N-TM /DMSO solution (**b**), respectively.

4) FeAzPc-8N-OB (Iron, [1,2,3,4,10,11,12,13,19,20,21,22,28,29,30,31-hexadecahydro-1,4,10,13,19,22,28,31-octakis(phenylmethyl)-37*H*,39*H*-tetraquinoxalino[6,7-*b*:6′,7′-*g*:6″,7″-*l*:6‴,7‴-*q*]porphyrazinato(2-)-κN^{37},κN^{38},κN^{39},κN^{40}]-, (*SP*-4-1)- (ACI), CAS: NA)

Synthetic route of FeAzPc-8N-OB is shown in **Figure S8**. The detailed procedure for preparation is outlined in the following steps:

Synthesis of 5,6,7,8-Tetrahydro-5,8-bis(phenylmethyl)pyrazino[2,3-b]pyrazine-2,3-dicarbonitrile: 4.0 g of 5,6-dichloropyrazine-2,3-dicarbonitrile, 5.0 g of *N*,*N*′-dibenzylethylenediamine, and 6.0 g of potassium carbonate were added to 60 mL acetonitrile and stirred for 20 hours at room temperature. Water was added and the precipitated solid was filtered off, the resulting solid was heated and dissolved in ethyl acetate, 300 mg of activated carbon was added and stirred for 10 minutes. The insoluble material was removed by celite filtration, the filtrate was concentrated, and the objective was obtained by crystallization from a mixture of ethyl acetate and isopropanol. Yield: 3.89 g (53.1 %).

Synthesis of FeAzPc-8N-OB: 104 mg of iron(II) chloride, tetrahydrate was added 20 mL of 1-

pentanol and stirred. After 10 mL of 1-pentanol was distilled off at atmospheric pressure, the mixture was cooled to 130 °C. After 4 hours, the mixture was cooled to room temperature and the precipitated solid was filtered off. After 4 hours, the mixture was cooled to room temperature and the precipitated solid was filtered out and washed with methanol. The resulting solid was purified by silica gel column chromatography (chloroform/methanol elution) to afford the desired product. Yield: 81 mg (10.6%).

Characterization: The structure of yielded compound was confirmed by MALDI-TOF-MS spectrum (**Figure S9**, left side plot). UV-Vis spectrum of its DMSO solution are shown in **Figure S9**, right side plot. MALDI-TOF-MS: Calculation: 1,521.557, Measured: m/z=1522.62 (the product was mainly fragmented) UV-Vis: λ_{max}= 538 nm.

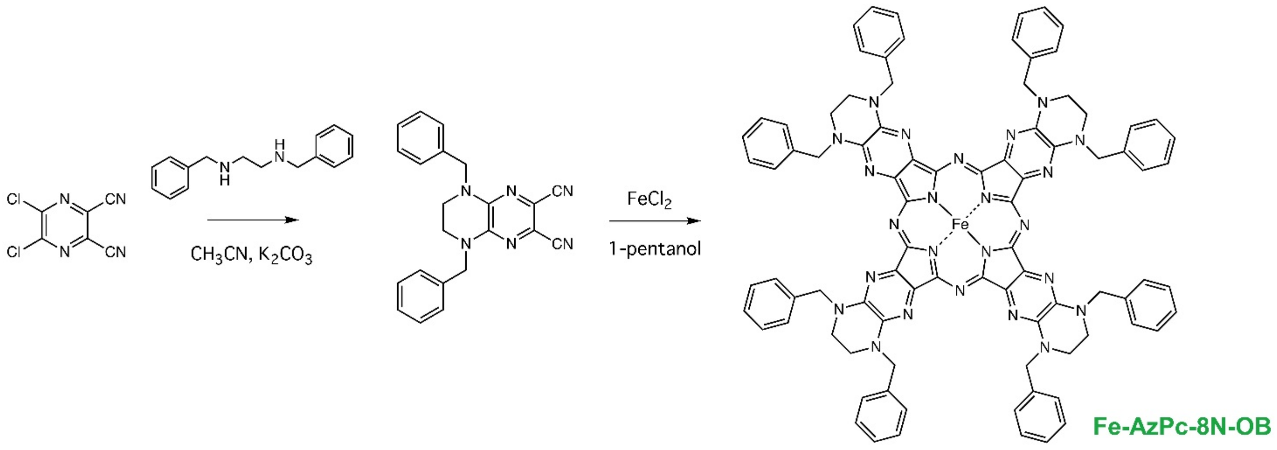

Figure S8. Synthetic route of FeAzPc-4N-OB.

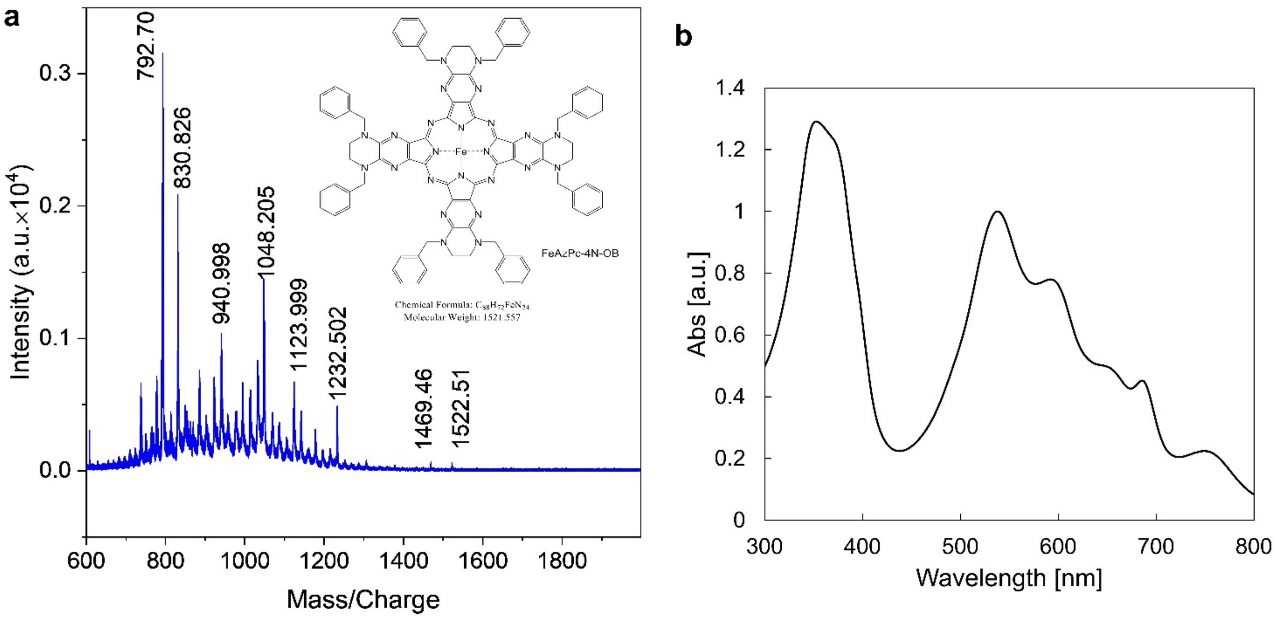

Figure S9. MALDI-TOF-MS spectrum of FeAzPc-8N-OB (**a**) and UV-Vis spectrum of FeAzPc-8N-OB /DMSO solution (**b**), respectively.

4. STEM-EDS analysis of catalysts

4.1 Common procedures

Catalyst preparation: First, 30 mg of Ketjen Black (KB, EC300J, Lion Specialty Chemicals, Co. Ltd., Tokyo) was dispersed in a dimethyl sulfoxide (DMSO) solution of 0.1 mg/mL metal-AzPc or FePc. The dispersion was sonicated with a homogenizer for 5 min and then suction-filtered to collect the samples. The samples were washed three times each with methanol and chloroform and then dried *in vacuo*.

STEM-EDS observation: A catalyst was dispersed in methanol with sonication and the dispersion was cast onto a Cu grid. The sample morphologies were observed by STEM (HD-2000, Hitachi Hitech, Japan) and STEM-EDS (JEM-ARM200F, JEOL Corp., Japan) equipped with a Cold Field Emission Gun and Cs probe corrector. STEM was performed at 80kV. Typical STEM images of catalyst-loaded KB are shown in Figure S9.

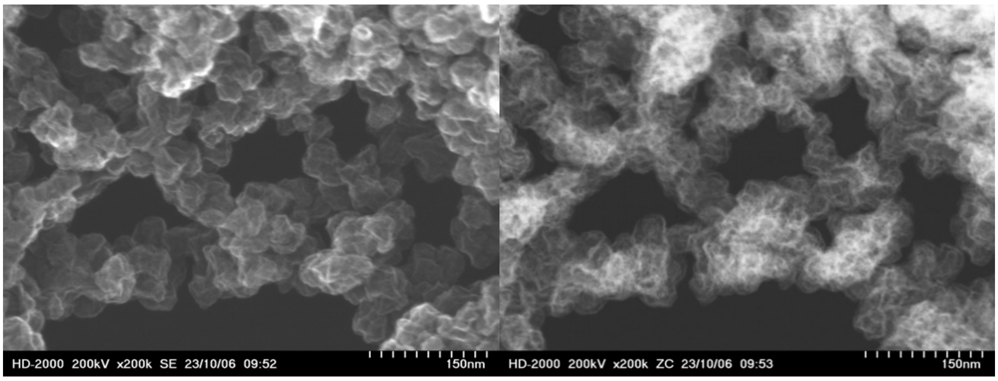

Figure S10. STEM SE (left) and Z-contrast (right) images of catalyst-loaded KB (FeAzPc-4N@KB), respectively.

TEM (TE) image, elemental mapping image, and EDS spectrum of catalyst-loaded KBs are shown below. STEM-EDS analysis results of FeAzPc-8N-8Me@KB, FeAzPc-4N-TM@KB, FeAzPc-4N-TS@KB, and FeAzPc-4N-OB@KB are also shown in **Figures S11-S14**, respectively. In each case, atomic-level Fe signals were observed, and the Fe signals were uniformly dispersed on the surface of KB, which supported the molecular adsorption of catalyst molecules onto the surfaces of KB particles.

1) FeAzPc-8N-8Me@KB

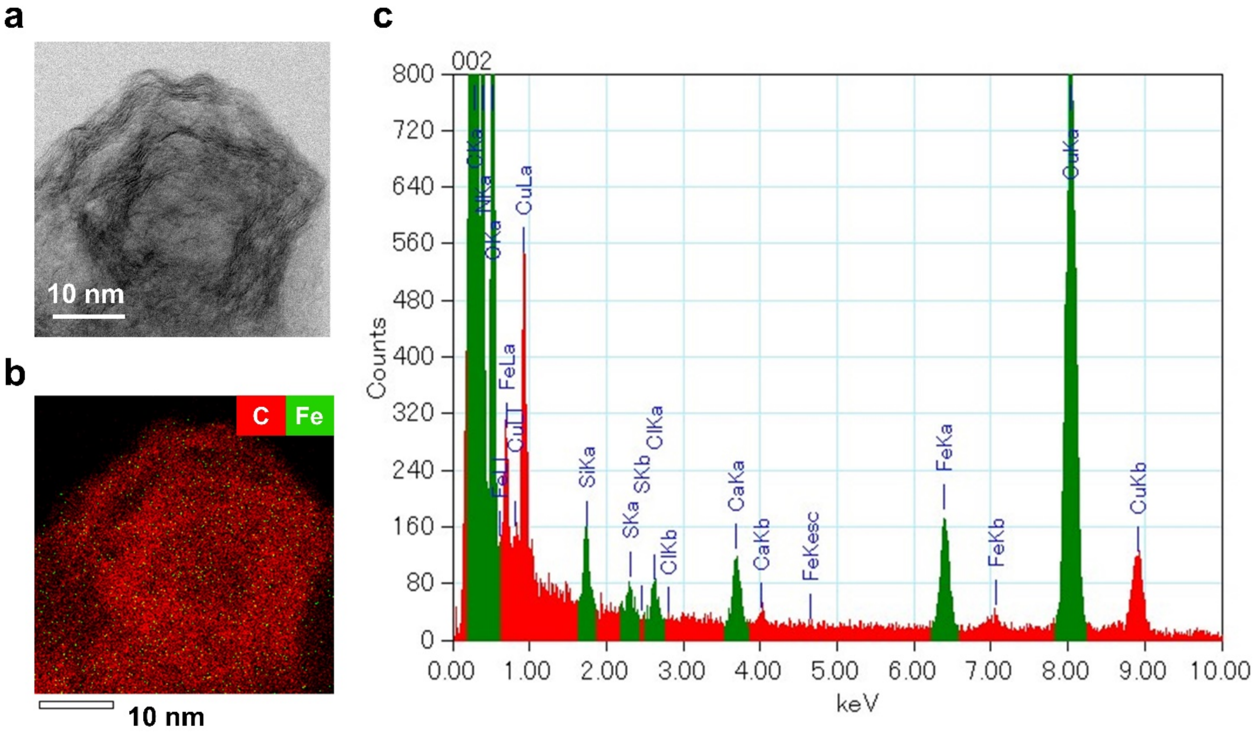

Figure S11. STEM (TE) image (**a**), elemental mapping image (**b**), and EDS spectrum (**c**) of FeAzPc-8N-8Me@KB, respectively.

2) FeAzPc-4N-TM@KB

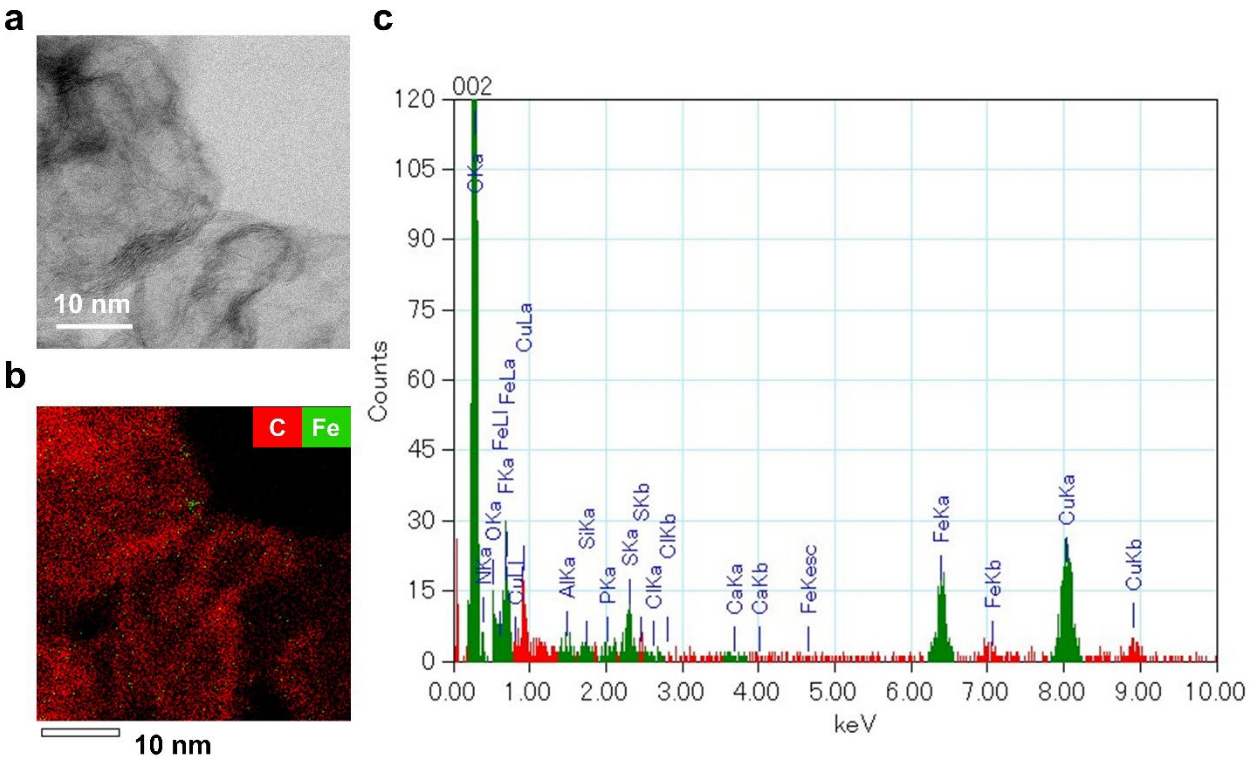

Figure S12. STEM (TE) image (a), elemental mapping image (b), and EDS spectrum (c) of FeAzPc-4N-TM@KB, respectively.

3) FeAzPc-4N-TS@KB

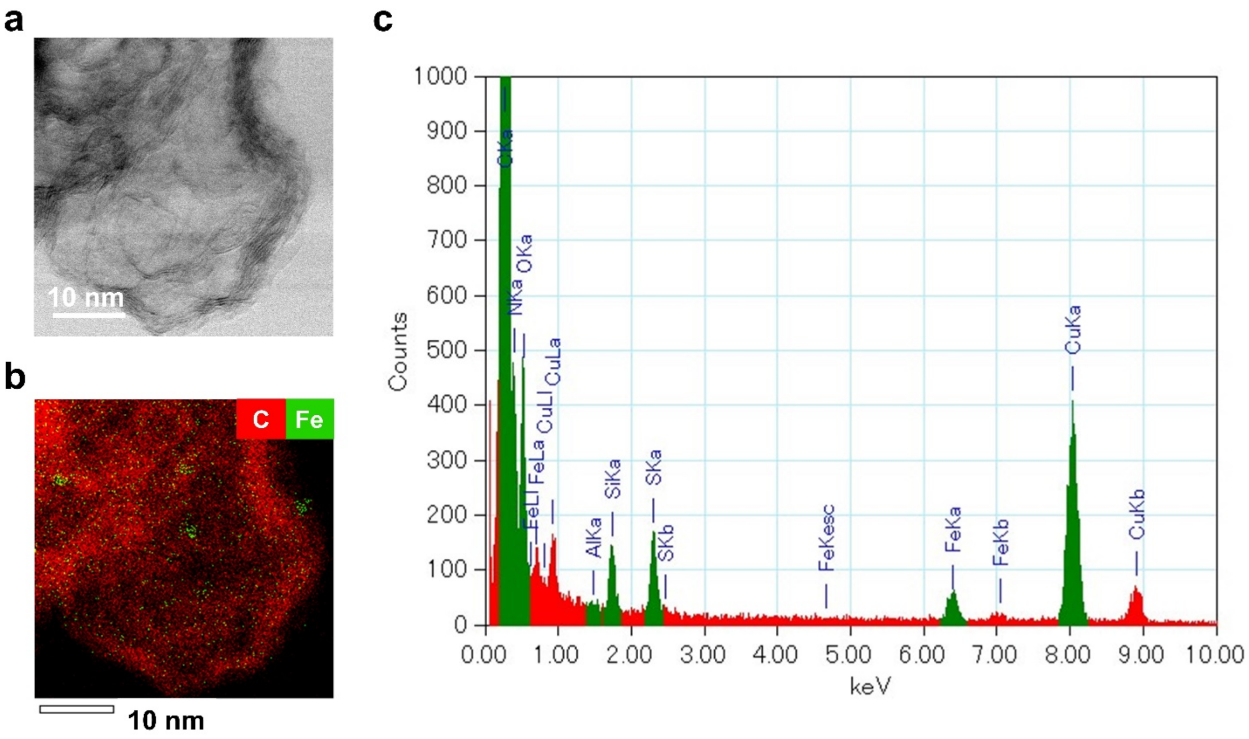

Figure S13. STEM (TE) image (a), elemental mapping image (b), and EDS spectrum (c) of FeAzPc-4N-OB@KB, respectively.

4) FeAzPc-4N-OB@KB

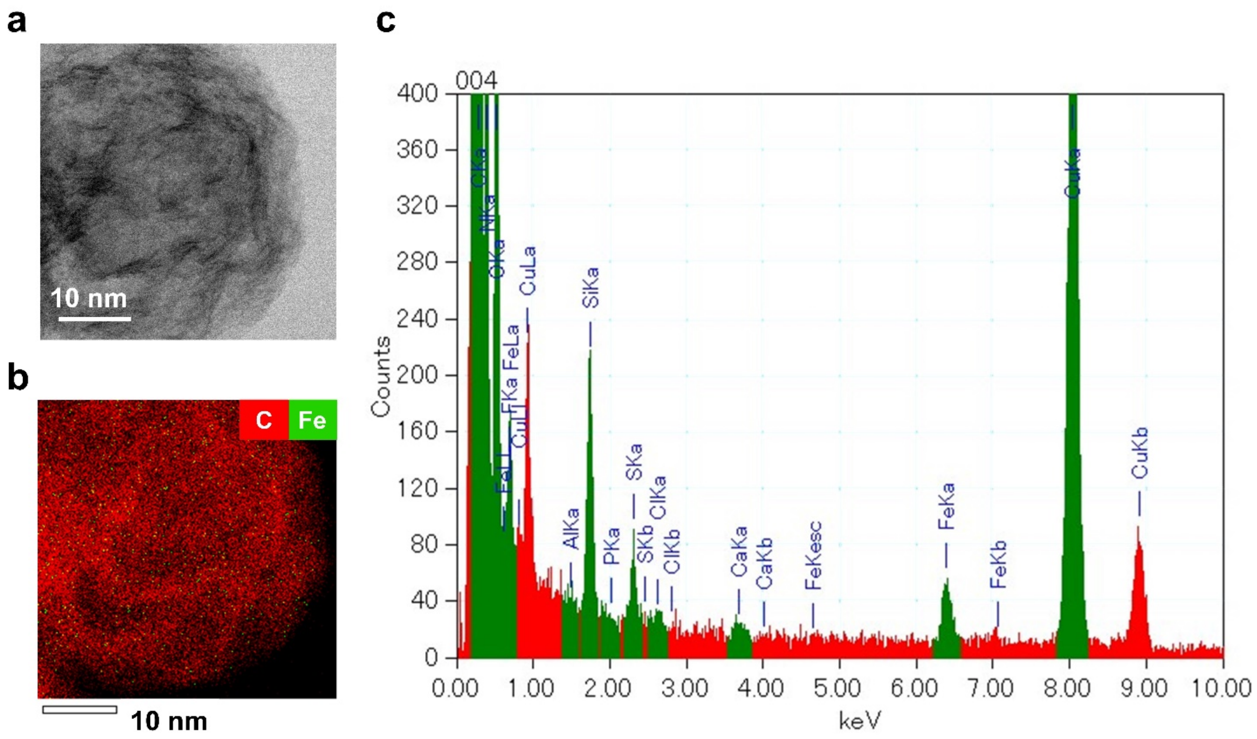

Figure S14. STEM (TE) image (a), elemental mapping image (b), and EDS spectrum (c) of FeAzPc-4N-OB@KB, respectively.

5. Pt/C ORR polarization curves for comparisons

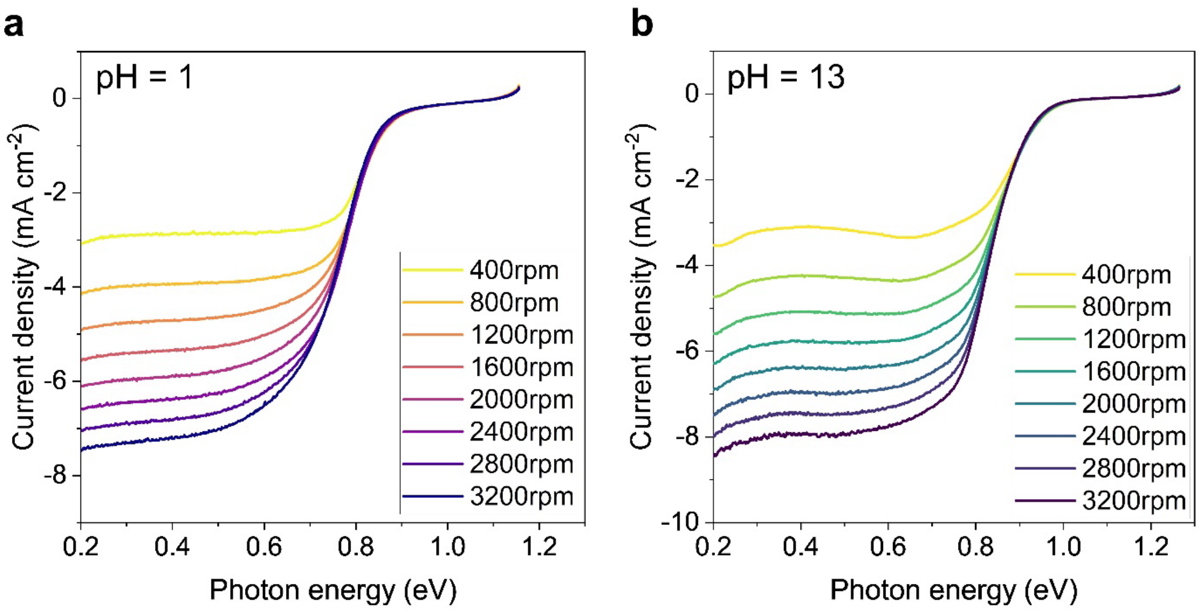

Figure S15. Experimental RDE polarization curves of Pt/C. (a) pH = 1; (b) pH = 13.

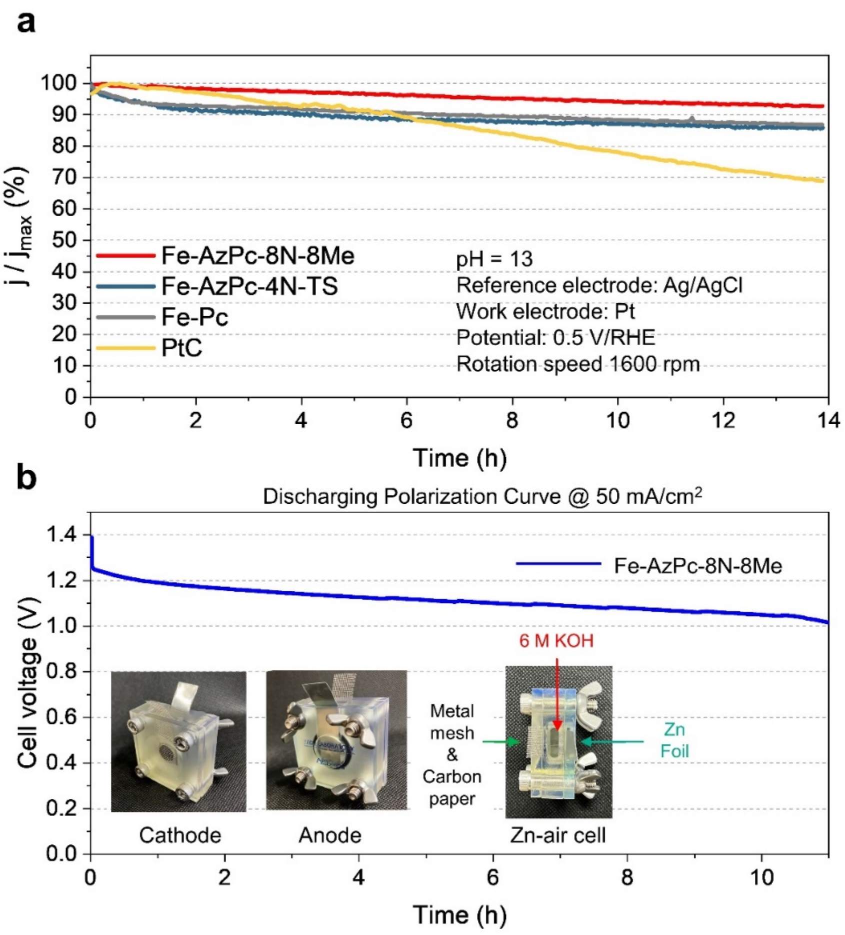

Figure S16. Stability tests of Fe-AzPc derivatives. (**a**) The durability of both Fe-AzPc-8N-8Me and Fe-AzPc-4N-TS, in comparison with Fe-Pc and Pt/C. (**b**) The durability of Fe-AzPc-8N-8Me in a Zn-air battery.
